# Transhepatic tract hemostasis using thermal-ablation after percutaneous portal vein access

**DOI:** 10.1259/bjrcr.20210080

**Published:** 2021-09-29

**Authors:** Mathilde Vermersch, Alban Denys, Florent Artru, Georgia Tsoumakidou, Nicolas Villard, Rafael Duran, Arnaud Hocquelet

**Affiliations:** 1 Department of Radiology, Lausanne University Hospital CHUV, Lausanne, Switzerland; 2 Department or Radiology, Lille University Hospital, Lille, France; 3 Department of Gastro-enterology, Lausanne University Hospital CHUV, Lausanne, Switzerland

## Abstract

**Objectives:**

Bleeding risk after percutaneous portal vein access procedures is not negligible. Various agents, coils and plug, have been used to minimize this risk, each with their own advantages and disadvantages. This study reports the results of coagulation using thermal-ablation (radiofrequency or microwave ablation) as an alternative to trans-hepatic puncture tract closure.

**Methods:**

Ten patients who benefited from portal vein recanalization or portal hypertension-relative bleeding complication embolization using percutaneous portal vein access and who underwent thermal-ablation of the puncture tract between December 30, 2019 and July 16, 2020 were included. Early efficiency and safety were evaluated using imaging (ultrasound and/or CT scan) and laboratory data (hemoglobin, hepatic function) at 24 h. Follow-up was performed until August 2020.

**Results:**

No bleeding from the puncture tract and no embolization-related complications were observed in all 10 patients at 24 h or during follow-up with median of 3 months (range 1–8 months), even in case of ascites or therapeutic coagulation.

**Conclusion:**

Thermal-ablation seems to be a safe, effective and rapid technique to avoid bleeding after percutaneous transhepatic direct portal vein access.

**Advances in knowledge:**

Thermal-ablation could be an alternative for transhepatic puncture tract closure especially for patients with high bleeding risk.

## Introduction

Treatment of portal vein stenosis/thrombosis^1–6^ or portal hypertension-related bleeding^7,8^ requires portal vein access, most often performed using percutaneous transhepatic portal vein approach.^9^ Removal of the sheath during the trans-hepatic approach can result in significant bleeding from the puncture tract, especially if an anticoagulant is used (recanalization)^10^ or if puncture tract closure is not performed.^9,11^ Embolization of the trans-parenchymal puncture tract has been described using various embolic agents including collagen,^3^ coils,^1,4,5,8^ Amplatzer vascular plug,^2^ Gelfoam,^5,8^ and N-butyl cyanoacrylate,^4,12^ each with their own drawbacks. Thermal-ablation is commonly used by interventional radiologists for liver tumor ablation with coagulation of puncture tract to avoid bleeding and tumor dissemination. The purpose of the present study was to report the initial results of coagulation of the trans-hepatic puncture tract using thermal-ablation (radiofrequency or microwave ablation) after percutaneous transhepatic direct portal vein access.

## Patients and methods

The Institutional Review Board approved this study (ID 2019–01409).

### Patients

Between December 30, 2019 and July 16, 2020, 10 patients were treated for portal vein stenosis/thrombosis (*n* = 9) or bleeding from portal hypertension (*n* = 1) using transhepatic portal vein access. Treatment by the percutaneous transhepatic approach was decided by interdisciplinary consensus. Written informed consent was obtained from all patients. All patients were retrospectively analyzed. The patients’ medical records, radiological records and images were evaluated. Patient characteristics are presented in Table 1. The platelet count varied from 54 to 416 x 10^9^/L and none of the patients had a prothrombin time under 65% or international normalized ratio upper than 1.2. Most patients received therapeutic doses of anticoagulation during and immediately after the procedure (80%).

**Table 1.** Patients characteristics and clinical outcomes

Patient Number	Sex	Age (years)	Intervention performed	Indication of treatment	Platelet Count (G/l)	Intervention time (min)	PT (%)	INR	Ascites	Portal Pressure (mmHg)	Portal branch Punctured	Per procedural anticoagulation (IU of heparin)	Post-operative anticoagulation	Thermo-ablation needle	Complication
1	Male	72	Portal recanalization	Pancreatic cancer with portal thrombosis Bleeding	415	80	75	1.2	No	15	VI	6600	Yes	Microwave NeuWave PR probe	No
2	Male	62	Portal recanalization	Pancreatic cancer with portal thrombosis Abdominal pain	236	80	90	1.1	No	14	VI	6600	Yes	Microwave NeuWave PR probe	No
3	Male	76	Portal recanalization	Pancreatic cancer with portal thrombosis Bleeding	229	50	85	1.1	No	9	VI	5000	Yes	Microwave NeuWave PR probe	No
4	Female	70	Portal recanalization	Cholangiocarcinoma with portal thrombosis Bleeding	185	170	85	1.1	No	.	III	1,0000	Yes	Microwave NeuWave PR probe	No
5	Male	75	Embolization of peristomial varices	Cirrhosis Peristomial varices bleeding	111	80	65	1.2	Yes	.	VIII	.	No	Microwave NeuWave PR probe	No
6	Male	74	Portal recanalization	Pancreatic cancer with portal thrombosis Abdominal pain	331	80	80	1.1	Yes	12	V	7500	Yes	Radiofrequency needle Covidien	No
7	Male	77	Portal recanalization	Pancreatic cancer Whipple with portal reconstruction Stenosis	302	70	90	1.1	Yes	26	V	1,0000	Yes	Radiofrequency needle Covidien	Bloody fluid in the ascites drain without active bleeding or hemodynamic disorder
8	Male	63	Splenic vein recanalization	Pancreatitis with splenic and portal vein thrombosis Gastric varices bleeding	54	150	65	1.2	No	27	VI	5000	Yes	radiofrequency Cluster needle Covidien	No
9	Female	73	Portal pressure measurement	Refractory ascites post Whipple	304	30	100	1	Yes	8	V	.	No	Microwave NeuWave PR probe	No
10	Male	57	Portal recanalization	Pancreatitis with portal stenosis Bleeding	136	75	90	1.1	No	11	V	5000	Yes	Microwave NeuWave PR probe	No

### Portal vein intervention

Based on the findings of contrast-enhanced CT of the abdomen performed before each intervention, the portal vein was accessed by a right transhepatic approach in nine patients (portal branch from segment V, VI or VIII) and by a left approach (portal branch from segment III) in one patient because of a prior right hepatectomy. An attempt was made in each patient to puncture a peripheral branch of the right portal vein under ultrasound guidance using a 21G needle (CHIBA, Boston Scientific, Natick, MA, USA). A guide wire was advanced into the portal vein and a 6F 25 centimeter size sheath (Super Arrow-Flex, Teleflex Medical) was inserted using the Neff percutaneous access set (Cook Medical, USA) by the Seldinger technique (diameter was chosen as small as possible to allow procedure). Intrahepatic portal pressure after procedure varied from 8 to 27 mm Hg. Therapeutic coagulation during and after the procedure was required in eight patients in whom splenic and portal vein (*n* = 1) or portal vein (*n* = 7) stenting was performed. One of the two remaining patients underwent embolization for portal hypertension-related bleeding and portography and pressure measurement was performed in another, with no significant stenosis. After treatment of the portal vein was complete, operator performed occlusion of the transhepatic puncture tract using thermal-ablation.

### Technique of puncture tract embolization

**Figure 1.**
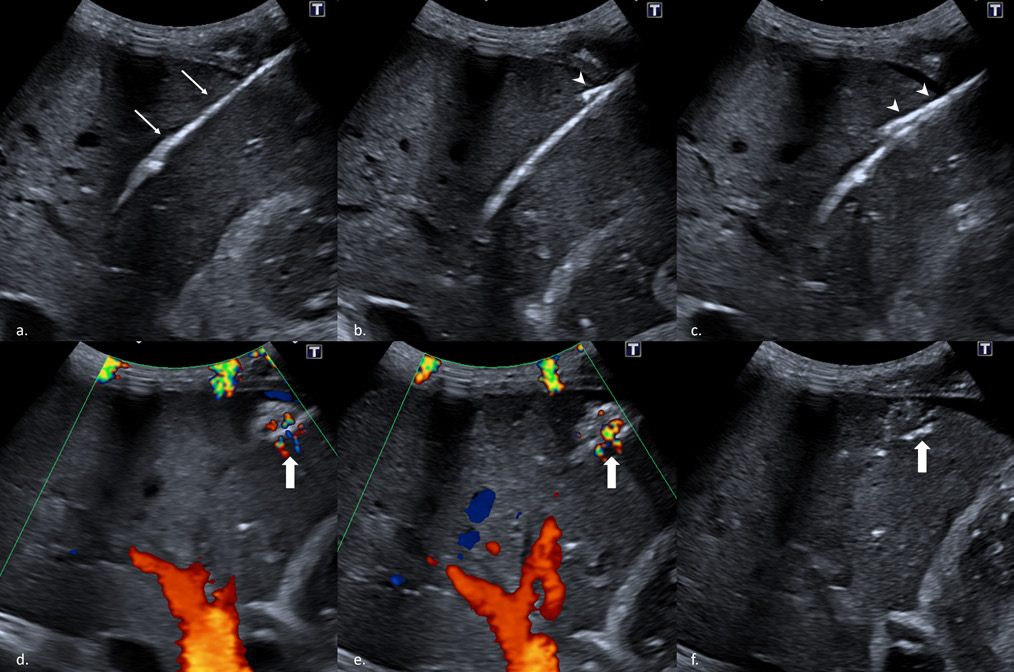
Puncture tract embolization technique. a.Ultrasound-guided identification of the puncture tract. The sheath is clearly visible (thin arrow). b and c. The microwave probe (arrowhead) is inserted along the puncture tract under ultrasound guidance and advanced 3 cm into the liver parenchyma. d and e. Ablation is performed under ultrasound guidance with 65W for 2 min, standard ablation parameters, to avoid ablation of soft tissue (thick arrow). f. Ultrasound is performed after removal of the material showing a triangular subcapsular ablation zone (thick arrow).

The puncture tract was coagulated using microwave (Neuwave PR probe 17G 15 cm, with emitting point at 1 cm proximal from the tip, Johnson and Johnson) in seven patients or with radiofrequency in the three remaining patients (Covidien Cool-Tip, with 2 cm active portion, Medtronic). The choice of microwave or radiofrequency was at the discretion of operator. The same operator who punctured the portal branch coagulated the puncture tract in all patients. A microwave or radiofrequency needle was introduced alongside the intrahepatic puncture tract under ultrasound guidance and advanced 2–3 cm into the liver parenchyma to close the capsular puncture site and limit the volume of ablated liver. Introduction of the thermal-ablation needle into the sheath, even if it is theoretically possible, was not retained because of the length of the sheath (too long, which would, therefore, have required an exchange over wire with risks of bleeding and loss of the path) and the coil-wire design (with risk of heat conduction). The sheath was then removed and thermal-ablation was performed using standard ablation parameters and stopped after one roll-off for radiofrequency and after 2 min at 65W for microwave. A power of 65W was chosen because only one shot was performed unlike a usual puncture tract ablation. An ultrasound was performed immediately after the procedure to check the intrahepatic and main portal vein patency and the absence of bleeding or subcapsular hematoma (Figure 1).

The day after the procedure, a formal abdominal ultrasound with hepatic Doppler or CT scan were performed by independent radiologist. Follow-up laboratory evaluation (hemogloblin and hepatic function) was also performed 24 h after procedure.

### Follow-up

The medical records (clinical and laboratory data) were evaluated to determine the technical and clinical success of the puncture tract embolization as well as local or general complications. Follow-up CT scans were evaluated to visualize the ablation zone.

## Results

Stenting for portal vein stenosis as well as for the embolization of portal hypertension-related varices was technically successful with no significant residual pressure gradient and no portal hypertension-related bleeding in all nine patients. The main portal vein was patent in all patients and no subcapsular hematomas were observed in the immediate post-operative ultrasound. No patient showed a decrease in hemoglobin level of more than 2 g.dl^−1^. Bloody fluid in the ascites drain was observed in one patient, without decrease in hemoglobin level or hemodynamic instability. A CT scan performed 6 h after the procedure did not find any active bleeding or hematoma.

**Figure 2.**
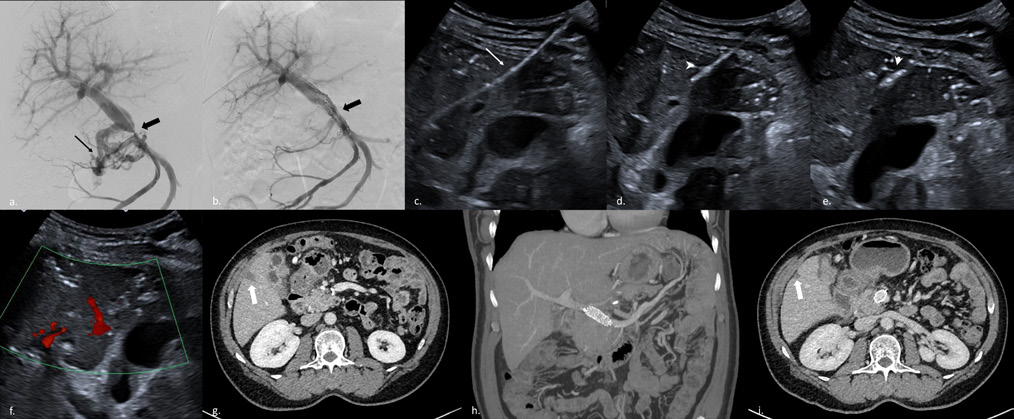
: Pre- and post-operative aspect a. Severe portal stenosis (black thick arrow) with collateral pathways (black arrow) b. Stent was deployed across the stenotic segment (black thick arrow) allowing satisfactory portal flow and disappearance of collateral pathways. c. Visualization of the puncture tract with the sheath (thin arrow) under ultrasound. d and e. Microwave probe (arrowhead) is inserted along the puncture tract and thermal-ablation is performed f. Ultrasound control using Doppler showed the absence of active bleeding and a patent portal vein g. First scan after intervention showed a sub capsular hypodense triangular patch corresponding to ablation zone (thick arrow) h. Second scan performed 5 months later with MIP reconstruction: patent stent without deviation route i. 5 months later, capsular retraction and decrease in the triangular patch (thick arrow).

There were no major biological complications. Significant transient cytolysis (five times above normal) occurred in one patient, which was entirely and spontaneously resolved in 3 days. Mild transient cytolysis (less than two times above normal) occurred in four patients and resolved spontaneously in 3 days. No cholestasis or hepatocellular insufficiency was observed. There were no infectious complications. Post-operative pain was mild-to-moderate and easily controlled by the usual analgesics. The ablation zone was easily visualized on CT scan as a subcapsular hypodense triangular patch. On subsequent scans, capsular retraction is seen with a decrease in the thermal-ablation zone (Figure 2)

One patient died during follow-up due to tumor progression with no complications from the intervention. The remaining nine patients were all still alive after a median follow-up of 3 months (1–8 months). No complications were observed in these patients.

## Discussion

The percutaneous transhepatic approach is the common access route for portal vein angioplasty or balloon occluded antegrade transvenous obliteration of varices.^9^ Without transhepatic tract closure, this approach is complicated by hemorrhage in up to  30% of cases.^9,11^ Bleeding from the puncture tract is usually venous and most likely a result of blood running from the punctured portal vein through the hepatic puncture tract into the peritoneal cavity. Several techniques have been described to prevent post-interventional bleeding from the puncture tract including collagen cylinders, coils, Amplatzer vascular plugs, N-butyl cyanoacrylate or Gelfoam. With these techniques, post-procedure bleeding risk was strongly decreased; however, there were some reports leading to surgical ligation.^2,5,8^ Moreover, embolic agents, when they are used by unexperienced radiologist, are not without risk: liquid agent migration, potentially leading to portal vein thrombosis, or incomplete tract embolization in case of high portal pressure; non-target embolization of coils when hepatic vein or bile duct have been traversed; transhepatic tract damage with oversized plug.^9^ Thermo-ablation, based on surgical cautery technique but without surgical approach, appears as an interesting alternative, this technology being commonly used and mastered by interventional radiologists for liver tumor ablation. The use of radiofrequency ablation to avoid bleeding complications after liver biopsy has already been described in *ex vivo* and *in vivo* experimental studies in pigs.^13,14^ These studies revealed a decrease in bleeding events even in a group receiving therapeutic coagulation. Recently, a letter was published reporting the emergency use of radiofrequency to stop active portal vein access bleeding^15^ in one patient after unsuccessful portal tract embolization.

Furthermore, with thermal-ablation, the ablation zone can be monitored under ultrasound guidance (as hyperechoic areas) avoiding peripheral portal vein thrombosis. Additional benefit, unlike other puncture tract closure techniques, this technique can be used in a second time, after sheath ablation, if ultrasound revealed bleeding.

Even if this technique have lots of advantages without major complications happened in our cohort, some disadvantages should be mentioned. Firstly, this technique remains expensive, especially in comparison with others techniques and should be considered as an alternative in patients with high bleeding risk. Secondly, the usual contraindications to thermal-ablation, like biliodigestive anastomosis or concomitant biliary obstruction, should apply to this technique to avoid complication (abscess). Thirdly, thermal-ablation zone should be monitored to avoid unnecessary parenchymal loss or subcutaneous tissues damage.

Some limitations of this study should be mentioned. Firstly, the study cohort was retrospectively selected and limited to a single institution. Secondly, the study is limited to a small sample size. However, it is a pilot study describing a new technique.

In conclusion, bleeding complications from transhepatic puncture tracts are rare but possible and systematic occlusion of the tract is recommended. Thermal-ablation (radiofrequency or microwave) seems to be a safe, effective and rapid technique to avoid bleeding in these patients. Thus, interventional radiologists should be aware of this possible alternative.

## Learning points

- Thermal-ablation could be used as an alternative to coil/gelfoam for transhepatic tract hemostasis
- Thermal-ablation is effective and safe for avoiding bleeding, especially in patients with high risk of bleeding.
